# Predictors of COVID-19 Vaccination among Veterans Experiencing Homelessness

**DOI:** 10.3390/vaccines9111268

**Published:** 2021-11-03

**Authors:** Michelle D. Balut, Karen Chu, June L. Gin, Aram Dobalian, Claudia Der-Martirosian

**Affiliations:** 1 Veterans Emergency Management Evaluation Center, U.S. Department of Veterans Affairs, North Hills, CA 91343, USA; Karen.Chu@va.gov (K.C.); June.Gin@va.gov (J.L.G.); Aram.Dobalian@va.gov (A.D.); Claudia.Der-Martirosian@va.gov (C.D.-M.); 2 Division of Health Systems Management and Policy, University of Memphis School of Public Health, Memphis, TN 38152, USA

**Keywords:** COVID-19 vaccines, vaccine hesitancy, SARS-CoV-2, homeless persons, Veterans, public health

## Abstract

Sufficient uptake of the COVID-19 vaccine is key to slowing the spread of the coronavirus among the most vulnerable in society, including individuals experiencing homelessness. However, COVID-19 vaccination rates among the Veteran homeless population are currently unknown. This study examines the COVID-19 vaccination rate among homeless Veterans who receive care at the U.S. Department of Veterans Affairs (VA), and the factors that are associated with vaccine uptake. Using VA administrative and clinical data, bivariate and multivariate analyses were conducted to identify the sociodemographic, health-related, and healthcare and housing services utilization factors that influenced COVID-19 vaccine uptake during the first eight months of the vaccine rollout (December 2020–August 2021). Of the 83,528 Veterans experiencing homelessness included in the study, 45.8% were vaccinated for COVID-19. Non-white, older Veterans (65+), females, those who received the seasonal flu vaccine, and Veterans with multiple comorbidities and mental health conditions were more likely to be vaccinated. There was a strong association between COVID-19 vaccination and Veterans who utilized VA healthcare and housing services. VA healthcare and homeless service providers are particularly well-positioned to provide trusted information and overcome access barriers for homeless Veterans to receive the COVID-19 vaccine.

## 1. Introduction

Infectious disease outbreaks often disproportionately impact those with underlying health conditions and individuals experiencing poverty, marginalization, and discrimination [1,2]. Amidst the current severe acute respiratory syndrome coronavirus 2 (SARS-CoV-2) pandemic, people who experience homelessness are particularly at risk of contracting and transmitting COVID-19 due to their transient nature and dependency on homeless shelters and other social service facilities, where overcrowding and high population turnover are common [3,4]. This population is also more likely to include people of color, those with chronic mental and physical conditions, and those with limited access to healthcare, all of which may lead to an increased risk of morbidity or mortality if they develop COVID-19 [4,5,6]. Despite the detailed statistics on COVID-19 deaths in the U.S., the mortality rate among individuals experiencing homelessness on a national level is largely unknown, yet it has been estimated that deaths in this population increased during the pandemic by 32% in Los Angeles, 54% in Washington, D.C., and 75% in New York City [7].

Long-term control of the pandemic largely depends on the sufficient uptake of the COVID-19 vaccine. The U.S. Centers for Disease Control and Prevention (CDC) issued guidelines advising that homeless persons residing in congregate facilities be among the first groups to be vaccinated [8], and ensuring that homeless Veterans receive the vaccine is important to the U.S. Department of Veterans Affairs’ (VA) effort in reducing the spread of the coronavirus [9]. The VA began distributing the Pfizer-BioNTech and Moderna COVID-19 vaccines to employees and Veterans in mid-December 2020 [10], and introduced the Janssen COVID-19 vaccine by Johnson & Johnson in early March 2021 [11]. Initial studies suggest that the early vaccination rollout period for Veterans was efficient, with 23% vaccinated by mid-March 2021 [12]. Among Veterans who were fully vaccinated for COVID-19 during this time, estimated vaccine effectiveness was 95% against infection and 91% against hospitalization [12]. As of late July 2021, approximately 51.5% of all Veterans receiving care at the VA have been vaccinated [13], compared to 64.3% of the U.S. adult population [14].

COVID-19 inoculation rates among the homeless population in the U.S., however, are currently unknown, as many state immunization registries do not track housing status [15,16], although it is estimated that unhoused people are being vaccinated at half the rate of the general population [15]. Vaccine uptake among people experiencing homelessness may be hindered by various factors including vaccine hesitancy, mistrust of authorities, mental or physical impairments, lack of transportation, or competing priorities, such as finding food or shelter [17,18]. Previous research examining the general vaccine hesitancy among homeless individuals have found high refusal rates even for longstanding illnesses such as influenza [19,20] and hepatitis [21]. However, few studies have explored the COVID-19 vaccination behaviors within the general homeless population, and to date, only one has examined the vaccine attitudes of homeless Veterans [22], who comprise a significant proportion of the U.S. homeless population. These studies reported that between 30–50% of homeless individuals were unwilling to get vaccinated [22,23]. Outside the U.S., studies in Italy [24] and France [25] found that 32.1% and 40.9% of unhoused persons were reluctant to receive the COVID-19 vaccine, respectively, suggesting the growing importance to better understand COVID-19 vaccine hesitancy and uptake behavior within this population.

This study examines the COVID-19 vaccination rates among homeless Veterans receiving care at the VA nationwide, as well as identifies the key sociodemographic, health-related, and healthcare and housing services utilization factors that influence vaccine uptake. By identifying these characteristics, the VA and other entities that provide care to homeless persons can effectively tailor health messaging for those individuals who may be vaccine hesitant.

## 2. Materials and Methods

### 2.1. Study Design and Data Sources

We conducted an observational cohort analysis of all Veterans who had an International Statistical Classification of Diseases, Tenth Revision, Clinical Modification (ICD-10-CM) homelessness diagnosis of Z59.0 (*n* = 83,528) in the one year prior to the COVID-19 vaccine rollout (14 December 2019–13 December 2020). The data for this study were obtained from the Veterans Health Administration (VHA) Corporate Data Warehouse (CDW), which is a national repository of data from VHA clinical and administrative systems. The study period was the first eight months of the vaccination rollout period (14 December 2020 through 1 August 2021).

### 2.2. Conceptual Framework

The study was guided by the Behavioral Model for Vulnerable Populations [26], which is a framework that identifies the predisposing, enabling, and need factors that influence health behaviors, including accessing services. This model builds on the Andersen Behavior Model of Health Services Utilization [27,28] by including specific constructs across each domain that are pertinent to the health of vulnerable populations. For example, the adapted model includes enabling factors such as family and community resources related to transportation and social services, and additional need factors such as evaluated health conditions [26]. This model has been previously applied in research with a variety of populations, including individuals experiencing homelessness [29,30,31,32,33], and has been used to examine Hepatitis B (HBV) [32] and human papillomavirus (HPV) [33] vaccination uptake, but has yet to be applied to analyze the predictors of COVID-19 vaccination.

### 2.3. Outcome Measure

The dependent variable was a binary COVID-19 vaccination indicator (0 = not vaccinated, 1 = vaccinated) during the eight-month study period. COVID-19 vaccinations were identified through Current Procedural Terminology (CPT) codes as specified by the U.S. Centers for Medicare and Medicaid Services (CMS) guide on “Coding for COVID-19 Vaccine Shots” [34], as well as inpatient bar code medication administration logs, patient immunization tables (which includes a field to record vaccinations that may have occurred outside or at non-VA locations, that were reported during subsequent patient care encounters), and the VA COVID-19 Shared Data Resource, which emerged as a new data resource containing information related to COVID-19 in and outside of the VA [35].

### 2.4. Statistical Analyses

**Figure 1:**
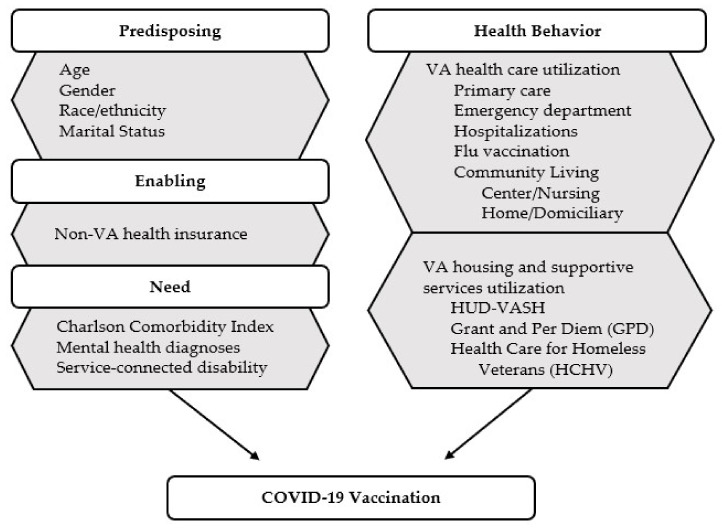
Predicting variables of COVID-19 vaccination among Veterans experiencing homelessness, guided by the Behavioral Model for Vulnerable Populations.

Bivariate analyses were used to descriptively compare homeless Veterans who received at least one dose of the COVID-19 vaccine to homeless Veterans who did not receive any dose of the COVID-19 vaccine during the eight-month vaccine rollout period. We used tests for the differences in categorical variables guided by the conceptual framework (see Figure 1 for the list of covariates). The predisposing demographic variables included in the analyses were age (18–44, 45–64, 65–74, 75+), gender (male, female), race/ethnicity (non-Hispanic White, non-Hispanic African American, Hispanic, non-Hispanic other, and unknown), and marital status (married, not married). The enabling variable was having health insurance other than VA insurance (yes/no). Need was approximated by the Charlson Comorbidity Index (CCI) (0, 1, 2+ comorbidities), which measures chronic disease burden to assess mortality risk [36], having any mental health diagnoses (yes/no) (i.e., alcohol use disorder, drug use disorder, posttraumatic stress disorder, psychotic disorders, depression, anxiety, schizophrenia, or bipolar disorder), and having any service-connected disability (yes/no). Examples of service-connected disability are varied, and include both mental and physical disabilities such as posttraumatic stress disorder, certain chronic and tropical diseases (e.g., multiple sclerosis, diabetes mellitus, and arthritis), disabilities related to in-service exposure to hazards (e.g., Agent Orange, Gulf War illnesses, radiation or herbicide exposure), etc. [37].

The health behaviors of interest were healthcare utilization during the study period across primary care (0–2, 3–5, 6+ visits) and emergency department (0–5, 6+ visits); having any hospitalizations (yes/no); having received an influenza vaccine during the 2019–2020 flu season (yes/no) (which could have been obtained within or outside the VA), and congregate living status in a VA Community Living Center, nursing home, or Domiciliary, which provide treatment and housing to geriatric Veterans and/or those with physical or mental disabilities or substance abuse disorders [38,39]. In addition, the utilization of VA’s housing and supportive services was examined (yes/no), including participation in the U.S. Department of Housing and Urban Development (HUD) and VA Supportive Housing (HUD-VASH) program, which provides subsidized vouchers for privately owned housing to eligible Veterans [40], the VA Grant and Per Diem (GPD) program, which issues funding to nonprofit organizations that provide transitional housing and additional social services to Veterans for up to two years [40], and the Health Care for Homeless Veterans (HCHV) program, which provides outreach, case management, and community-based residential services to chronically homeless Veterans [41].

Lastly, we used multivariable logistic regression models to further explore which variables from Figure 1 were associated with receiving at least one dose of the COVID-19 vaccine. The multivariable analyses excluded individuals with missing data on one or more study variables. The logistic model specification took into account the geographical locations of VA medical centers by controlling the clustering effect of city and state. *p* values were 2-sided and considered statistically significant at less than 0.05. All analyses were computed using Stata 15.1 (StataCorp, College Station, TX, USA).

## 3. Results

Among the study population, 45.8% (*n* = 38,269) were vaccinated during the study period. Table 1, column 1 (% population) displays the percentage of the study population for each patient characteristic during the study period. In terms of overall patient characteristics for the analytic sample of Veterans experiencing homelessness, 24.1% were between the ages of 18–44, 52.5% were 45–64 years old, 19.8% were ages 65–74, and 3.6% were 75 years of age or older, 90.4% male, 47.6% non-Hispanic White, 37.1% non-Hispanic African American, 6.7% Hispanic, 5.7% unknown race/ethnicity, and the remainder non-Hispanic Asian, American Indian, Alaskan Native, Hawaiian or Other, 13.9% married, and 36.0% had non-VA health insurance coverage. In terms of medical conditions, 84.6% had a mental health diagnosis, 49.6% had a service-connected disability, and the distribution of the Charlson score was as follows: 46% 0, 20.4% 1, 33.6% 2+. For utilization of care, 29.3% were vaccinated for the seasonal flu (2019–2020 flu season), 6.0% lived in a VA treatment center or nursing home, and the distribution for primary care visits included: 37.1% 0–2 visits, 25.5% 3–5 visits, 37.4% 6+ visits; emergency department visits: 89.9% 0–5 visits, 10.1% 6+ visits; and 33.7% had at least one hospitalization during the study period. With regard to HUD-VASH, HCHV, and GPD programs, the distribution of Veterans experiencing homelessness who participated in these programs were: 48.6%, 25.2%, and 9.4%, respectively.

Table 1, column 2 (% COVID vaccinated) displays the percentage of the study population that was vaccinated for COVID-19 for each patient characteristic. According to these unadjusted findings, younger Veterans experiencing homelessness compared to older Veterans experiencing homelessness were less likely to get vaccinated: 28.1% for ages 18–44, 49.2% for ages 45–64, 56.7% for ages 65–74, and 55.7% for ages 75 or older. Females (46.6%) compared to males (37.8%), and patients not married (46.6%) compared to married patients (42.1%) were more likely to get vaccinated. Non-Hispanic Whites (45.5%) were less likely to get vaccinated than non-Hispanic African Americans (47.3%) and Hispanics (48.2%). Veterans experiencing homelessness with non-VA health insurance coverage (54.4%) were more likely to get vaccinated, compared to VA-only insured Veterans experiencing homelessness (41.0%). Those with any service-connected disability were marginally less likely to get vaccinated (45.3% vs. 46.4%). Veterans with any mental health conditions (47.2% vs. 38.3%) and those with a higher Charlson score (2+ score 58.7% vs. 0 score 35.1%) were more likely to get vaccinated. In terms of healthcare utilization, those with more primary care visits (58.0% 6+ visits vs. 32.6% 0–2 visits), more emergency department visits (56.1% 6+ visits vs. 44.7% 0–5 visits), and any hospitalization (52.0% vs. 42.7%) were more likely to get vaccinated. Veterans who received their flu shot for the 2019–2020 flu season (63.6% vs. 38.4%) and those living in a VA treatment center or nursing home (74.7% vs. 44.0%) were more likely to get vaccinated. Lastly, Veterans who participated in a VA housing or supportive service program were more likely to get vaccinated than those who were not enrolled in a program: HUD-VASH (51.5% vs. 40.5%), HCHV (53.2% vs. 43.3%), GPD (60.7% vs. 44.3%). All bivariate comparisons were statistically significant at *p* < 0.001.

The results of the logistic regression analysis are summarized in Table 2. There is evidence of adjusted associations for demographic, health conditions, and healthcare and housing service utilization variables with COVID-19 vaccination. Hispanics and non-Hispanic African Americans had a higher percentage of individuals who were vaccinated for COVID-19 than non-Hispanic Whites. The vaccination percentage was 5.0 points higher for Hispanics, and 1.3 points higher for non-Hispanic African Americans than non-Hispanic Whites. Regarding age, vaccination percentages among 65–74 and 75 years of age and older were higher than among younger Veterans. Male Veterans experiencing homelessness had a lower vaccination rate compared to women (3.4 points lower). Vaccination among those with any service-connected disability was higher than the no disability group (2.3 points higher). In terms of primary care utilization, those with a higher number of visits (6+) had a significantly higher vaccination rate compared to fewer visits (0–2). Similarly, vaccination among those who lived in a VA treatment center or nursing home was dramatically higher than those who did not reside in one of these facilities (26.6 percentage points higher). COVID-19 vaccination among those receiving flu shots was dramatically higher than among those not receiving flu shots. Regarding the use of VA housing and supportive services, those in HUD-VASH, HCHV, or GPD programs had higher vaccination rates compared to Veterans experiencing homelessness who were not enrolled in any of these programs. The percentage point differences were as follows: HUD-VASH (9.3), HCHV (4.4), and GPD (12.3), where GPD had the highest percentage point difference.

## 4. Discussion

Concerns over the safety, efficacy, and novelty of the COVID-19 vaccines have led to a sizable proportion of the U.S. population being reluctant to vaccination. This study found that 45.8% of Veterans experiencing homelessness across the U.S. have received at least one dose of the COVID-19 vaccine, which is below the vaccination rates for both the general adult (64.3%) and overall Veteran populations (51.5%). To our knowledge, this is the largest study of homeless persons’ uptake of the COVID-19 vaccine to date.

Vaccine hesitancy is often linked to issues such as confidence, complacency, and convenience [42,43,44]. Additionally, complex factors such as poverty, systemic racism, health inequalities, and access barriers can hinder vaccine confidence and uptake [42,45]. Unhoused persons experience most, if not all, of these obstacles. Given their competing needs for housing, employment, and food, people experiencing homelessness often have difficulty prioritizing health needs [46], and thus might be expected to have lower vaccination uptake. This study found that enrollment in VA healthcare, housing, and supportive services mitigated this effect, increasing COVID-19 vaccine uptake among homeless Veterans. The likelihood of being vaccinated was significantly higher among those with multiple primary care visits and those housed in a treatment center or nursing home affiliated with or located on the VA campus, which is likely the result of both convenient access to care and having a trusted relationship with their VA healthcare provider. Previous literature found that routine access to medical care and recommendations from a healthcare provider were strong predictors of influenza vaccination among homeless individuals [19,20] and older Veterans [47]. Additionally, healthcare workers and social service providers have been cited as the most trusted sources of information on COVID-19 vaccines among homeless Veterans [22] and HIV-positive Black Americans [48]. As found in prior studies examining the association between willingness to receive the seasonal influenza and influenza A (H1N1) vaccines [49,50], Veterans in this study who received an influenza vaccine during the 2019–2020 flu season were also more likely to get a COVID-19 vaccine, indicating a higher acceptance of vaccines among flu shot recipients.

This study also found that Veterans who were enrolled in VA housing or supportive services, particularly GPD transitional housing programs, were dramatically more likely to be vaccinated. These programs provide a vital safety net for Veterans who lack basic resources, particularly during disasters which often disproportionately affects this population and disrupts access to care and services [51,52]. Gin et al. (2021) found that Veterans enrolled in GPD programs reported no access barriers to receiving a COVID-19 vaccine, as GPD staff members assisted in scheduling appointments for Veterans, providing transportation to vaccine clinics, or facilitating vaccination onsite. GPD staff also offered trusted relationships that encouraged and normalized vaccination uptake, possibly reducing hesitancy [22]. By providing a safe living space and access to regular healthcare, housing and social service programs have been shown to improve health behaviors and outcomes [20,32,46,53,54,55]. The fact that homeless Veterans in this study are being vaccinated at rates comparable to other Veterans (45.8% vs. 51.5%) may indicate that the VA’s programs are providing the support necessary to overcome barriers to care that people experiencing homelessness regularly face. Healthcare workers and homeless service providers are thus key to removing barriers and providing convenient access to the COVID-19 vaccine and improving uptake among Veterans experiencing homelessness. Further efforts and additional funding could potentially increase the outreach and enrollment of more homeless Veterans into these existing programs and improve both COVID-19 vaccination and health outcomes beyond the current pandemic.

Contrary to earlier trends of racial disparities to COVID-19 vaccine equity and acceptance, African American and Hispanic Veterans experiencing homelessness were more likely to be vaccinated, which supports the results of a study by the Kaiser Family Foundation that white Americans were more likely to refuse the vaccine [56]. These findings may be a result of increased outreach efforts to racial/ethnic minorities by the VA, increased risk of morbidity and mortality from COVID-19 among these groups, and/or may reflect greater dysfunction and medical non-adherence often seen among white homeless people [32,57,58]. In addition, older Veterans, those with multiple comorbidities, those with any mental health conditions, and those with multiple primary care visits, all likely indicators of medically complex patients, were more likely to be vaccinated. These groups were disproportionately affected by COVID-19 and were thus among the first to be offered the vaccine. Furthermore, studies have shown that individuals with greater health risks were more open to vaccination to avoid further health problems and those who were healthy/had fewer comorbidities were less open to vaccination, as they likely saw no need for additional prevention measures [32,59].

Despite the devastating impact of COVID-19 on the Veteran community, with over 15,000 known deaths [60], COVID-19 vaccine hesitancy has been reported to be higher among active-duty service members than the general population [61,62], suggesting that military culture and identity may influence vaccination decisions. One study examining COVID-19 vaccination behaviors and attitudes of homeless Veterans in GPD transitional housing found that military-specific experiences, such as adverse reactions to mandated vaccines, perceived substandard care, and harmful environmental exposures, created distrust toward the government and led to a refusal of the vaccine in that population [22]. Extant literature similarly found that military training and a sense of self-reliance may result in an unwillingness to follow health authorities’ recommendations [63,64]. Furthermore, studies have found that both Veterans [65] and the general homeless population [66,67] often have high levels of distrust in authority. Therefore, public health messaging should better utilize trusted sources, such as healthcare workers, homeless service providers, or Veteran peers, to provide information about the COVID-19 vaccine.

### Limitations

The sample size of 83,528 homeless Veterans is greater than what was previously reported by HUD (37,252 Veterans) [68]. However, those numbers are based on data that was collected in January 2020, prior to the COVID-19 outbreak, and many 2020–2021 homeless Point-in-Time counts were canceled because of COVID precautions [15]. Homelessness was rising across the country even before COVID-19 [69], and the pandemic and resulting economic recession likely exacerbated the homelessness crisis, with one study predicting an increase in general homelessness in the U.S. by 45% within a year [70]. Second, this study only included Veterans who received care from the VA and were identified as homeless by their clinicians between December 2019–December 2020. The dataset was limited and could not compare COVID-19 vaccination between homeless Veterans with non-homeless Veterans. Therefore, the findings may not be generalizable to non-homeless Veterans or homeless Veterans who do not receive care from the VA, as this latter group may experience additional barriers to receiving vaccinations and may be more distrustful of the government and other sources of healthcare information. Lastly, the electronic healthcare data had incomplete information about which vaccine was administered, and it had no data on Veterans’ COVID-19 diagnoses prior to vaccination, whether there were any adverse reactions to the COVID-19 vaccines, nor any information on COVID-19 mortality, all of which may be predictors of COVID-19 vaccination, and should be explored in future studies.

## 5. Conclusions

Improving vaccination rates will alleviate transmission concerns in homeless housing programs and mitigate the health effects of COVID-19 for those who are at higher risk for severe illness. As the coronavirus’s highly transmissible Delta variant has become the most prevalent strain in the U.S., and is almost exclusively affecting people who have not been vaccinated [71], it is increasingly vital to understand which factors are associated with COVID-19 vaccination among people experiencing homelessness so that public health messaging can be tailored for groups that are more vaccine hesitant, such as younger Veterans, white men, and those who do not regularly receive medical care or housing services. Most importantly, VA healthcare and homeless service providers are particularly well-positioned to provide trusted information and overcome access barriers for homeless Veterans to receive the COVID-19 vaccine, and ultimately slow the spread of the virus among the most vulnerable in society.

## Figures and Tables

**Table 1:** Patient characteristics of Veterans experiencing homelessness (overall) and by COVID-19 vaccination status.

Patient Characteristics		% Population	% COVID Vaccinated
		*N* = 83,528	45.8%
Age Categories:	18–44	24.1%	28.1%
45–64		52.5%	49.2%
65–74		19.8%	56.7%
75+		3.6%	55.7%
Gender	Male	90.4%	37.8%
Female		9.6%	46.6%
Race/Ethnicity	Hispanic or Latino	6.7%	48.2%
Non-Hispanic Other		2.8%	44.0%
Non-Hispanic African American		37.1%	47.3%
Non-Hispanic White		47.6%	45.5%
Unknown		5.7%	38.7%
Marital Status	Married	13.9%	42.1%
Not Married		86.1%	46.6%
Health Insurance (non-VA)	Yes	36.0%	54.4%
No		64.0%	41.0%
Flu Shot 2019–2020	Yes	29.3%	63.6%
No		70.7%	38.4%
Any Service-Connected Disability	Yes	49.6%	45.3%
No		50.4%	46.4%
Community Living Centers/Nursing Homes/Domiciliary	Yes	6.0%	74.7%
No		94.0%	44.0%
Any Mental Health Diagnoses	Yes	84.6%	47.2%
No		15.4%	38.3%
Charlson Comorbidity Score	0	46.0%	35.1%
1		20.4%	48.8%
2+		33.6%	58.7%
Primary Care Visits	0–2	37.1%	32.6%
3–5		25.5%	47.2%
6+		37.4%	58.0%
Emergency Department Visits	0–5	89.9%	44.7%
6+		10.1%	56.1%
Any Hospitalization	Yes	33.7%	52.0%
No		66.3%	42.7%
HUD-VASH	Yes	48.6%	51.5%
No		51.4%	40.5%
Health Care for Homeless Veterans (HCHV)	Yes	25.2%	53.2%
No		74.8%	43.3%
Grant and Per Diem (GPD)	Yes	9.4%	60.7%
No		90.6%	44.3%

Note: For categorical variables, chi-square test was conducted. All comparisons were statistically significant *p* < 0.001. COVID-19 vaccination at the VA (nationally) 14 December 2020–1 August 2021.

**Table 2:** Logistic regression predicting COVID-19 vaccination among Veterans experiencing homelessness.

Patient Characteristics		Adjusted % COVID Vaccinated	Non-Reference vs. Reference (ref) Difference in % COVID Vaccinated (95% CI)
Age Categories:	18–44 (ref)	34.3%	N/A
45–64		48.2%	13.8% (12.7%, 15.0%)
65–74		52.3%	17.9% (16.4%, 19.4%)
75+		51.7%	17.4% (15.0–19.7%)
Gender	Male	42.8%	−3.4% (−4.5%, −2.4%)
Female (ref)		46.3%	N/A
Race/Ethnicity	Hispanic or Latino	50.2%	5.0% (0.3%, 6.8%)
Non-Hispanic Other		47.0%	1.8% (−1.3%, 4.9%) *ns*
Non-Hispanic African American		46.4%	1.3% (0.2%, 2.3%)
Non-Hispanic White (ref)		45.2%	N/A
Unknown		44.0%	−1.1% (−2.9%, 0.5%) *ns*
Marital Status	Married	45.1%	−1.0% (−2.0%, 0.0%) *ns*
Not Married (ref)		46.1%	N/A
Health Insurance (non-VA)	Yes	48.4%	3.8% (2.9%, 4.7%)
No (ref)		44.5%	N/A
Flu Shot 2019–2020	Yes	58.3%	17.5% (16.6%, 18.5%)
No (ref)		40.7%	N/A
Any Service-Connected Disability	Yes	47.1%	2.3% (1.7%, 2.9%)
No (ref)		44.8%	N/A
Community Living Centers/Nursing Homes/Domiciliary	Yes	70.9%	26.6% (24.5%, 28.7%)
No (ref)		44.4%	N/A
Any Mental Health Diagnoses	Yes	46.2%	1.6% (0.3%, 2.8%)
No (ref)		44.6%	N/A
Charlson Comorbidity Score	0 (ref)	42.9%	N/A
1		46.6%	3.8% (2.7%, 4.7%)
2+		49.4%	6.5% (5.6%, 7.4%)
Primary Care Visits	0–2 (ref)	38.5%	N/A
3–5		47.0%	8.5% (7.7%, 9.4%)
6+		52.3%	13.9% (12.8%, 15.0%)
Emergency Department Visits	0–5 (ref)	45.9%	N/A
6+		46.1%	0.1% (−1.2%, 1.5%) *ns*
Any Hospitalization	Yes	46.1%	0.1% (−0.7%, 1.1%) *ns*
No (ref)		45.9%	N/A
HUD-VASH	Yes	50.7%	9.3% (8.0%, 10.5%)
No (ref)		41.4%	N/A
Health Care for Homeless Veterans (HCHV)	Yes	49.3%	4.4% (3.3%, 5.5%)
No (ref)		44.8%	N/A
Grant and Per Diem (GPD)	Yes	57.1%	12.3% (10.6%, 14.1%)
No (ref)		44.8%	N/A

Note: All comparisons were statistically significant *p* < 0.001, except for “*ns*”, which indicates not significant. COVID-19 vaccination at the VA (nationally) 14 December 2020–1 August 2021.

## Data Availability

The datasets generated and/or analyzed during the current study are not publicly available. They are available from the corresponding author on reasonable request, subject to approval from the ethics committee that approved the study.
